# Enhanced chemosensitization of anoikis-resistant melanoma cells through syndecan-2 upregulation upon anchorage independency

**DOI:** 10.18632/oncotarget.18616

**Published:** 2017-06-27

**Authors:** TingTing Tseng, WuChing Uen, JenChih Tseng, ShaoChen Lee

**Affiliations:** ^1^ School of Medicine, Fu Jen Catholic University, New Taipei City 242, Taiwan; ^2^ Department of Hematology and Oncology, Shin Kong Wu Ho-Su Memorial Hospital, Taipei City 111, Taiwan

**Keywords:** melanoma, anchorage independency, syndecan-2, chemosensitivity

## Abstract

Syndecan family proteins are heparan sulfate proteoglycans, which involved in various cellular activities and associating with metastatic potential and chemosensitivity of tumor cells. Melanoma is one of malignant tumors with poor prognosis upon metastasis. Previously, we had shown that melanoma cells remained survived under cell detachment, which was similar to the initial steps of tumor metastasis. Downregulation of syndecan-1 and upregulation of syndecan-2 in melanoma A375 cells were observed by different suspension conditions. Specific gene alterations also increased melanoma malignancy under anchorage independency. Thus, we would like to investigate in further the role of specific gene alteration, so that it could be used to develop novel strategy to treat melanoma.

In this paper, we found that syndecan-2 expression level as well the kinase phosphorylation levels increased upon anchorage independency. The pathway to regulate syndecan-2 expression shifted from PKCα/β-dependent under adhesion into PKCδ-dependent under cell suspension. Manipulation of syndecan-2 expression showed that PI3K and ERK phosphorylation as well the migratory ability increased with increased syndecan-2 expression level. In addition, suspended melanoma cells were more sensitive to chemoagents, which correlated with syndecan-2 overexpression, PI3K and ERK activations, serum level, and the presence of glycosaminoglycans.

In conclusion, we showed upregulation of syndecan-2 in anoikis-resistant melanoma cells enhanced chemosensitivity through PI3K and ERK activation. This observation would support and refine the strategy of adjuvant chemotherapy to overcome metastatic melanoma.

## INTRODUCTION

Melanoma is aggressive skin cancer with high morbidity, high mortality, and poor prognosis for its highly invasive characteristics. Surgical removal of melanoma *in situ* is the major management [1], while it is difficult to remove completely for its re-occurrence with distant metastasis [2]. Detection and removal of micrometastases in sentinel lymph node and the following adjuvant therapy might prevent relapse of metastatic melanoma but some debates remained [3, 4]. Metastasis of tumor cells could be divided into several stages and characteristics distinct from their original tissue sources, including transformation to escape from primary tumors, intravasation into circulation system, dissemination in anchorage-independent / prolonged survival, and lodging at the secondary site for successive angiogenesis process [5–7].

Detachment of tumor cells from extracellular matrix and survival under anchorage-independency was recognized as initial step of tumor metastasis [8]. Detachment of normal cells causes cell apoptosis through anoikis signaling [9, 10], while the anoikis resistance of tumor cells ensured their survival and metastasis [11]. It was suggested that anchorage-independent growth promoted oncogenic transformation through hyperactive signaling pathways associated with epithelial-mesenchymal transition [8, 12–14]. Many studies had focused on the change of protein expression for malignant melanoma cells under anchorage-independency [2, 15]. However, the understanding how these changes were contributive to alternative phenotypes of tumor cells was limited.

Proteoglycans are major components of extracellular matrix s in different species, tissues, and cells. Of all the proteoglycans, heparan sulfate proteoglycans are exclusively important due to their involvement in several physiological processes or human diseases, including cell proliferation, differentiation, adhesion, migration, and tumor metastasis [16–18]. Syndecan superfamily contains four members of transmembrane-type proteoglycans with homologous cytoplasmic domains but distinct extracellular domains. Their protein domains contact with extracellular components and function as acceptors for various growth factors, chemokines, and adhesion molecules. Syndecan-2 (SDC2) had been shown to be associated with increased migration and invasion of melanoma cells [19] and melanin synthesis [20]. SDC2 was known to induce cell apoptosis in osteosarcoma through JNK signaling [21]. Overexpression of SDC2 sensitized human osteosarcoma cells to apoptosis induced by chemoagents [22], as well the fibroblast cells to apoptosis induced by serum-withdrawal [23].

In this paper, we characterized the upregulation of SDC2 in melanoma cells and investigated their functional role in cell migration, signaling, and chemosensitivity. That would be beneficial to develop one novel strategy to eradicate melanoma dissemination.

## RESULTS

### Increased SDC2 expression and activation of signaling kinase in suspended melanoma cells

**Figure 1:**
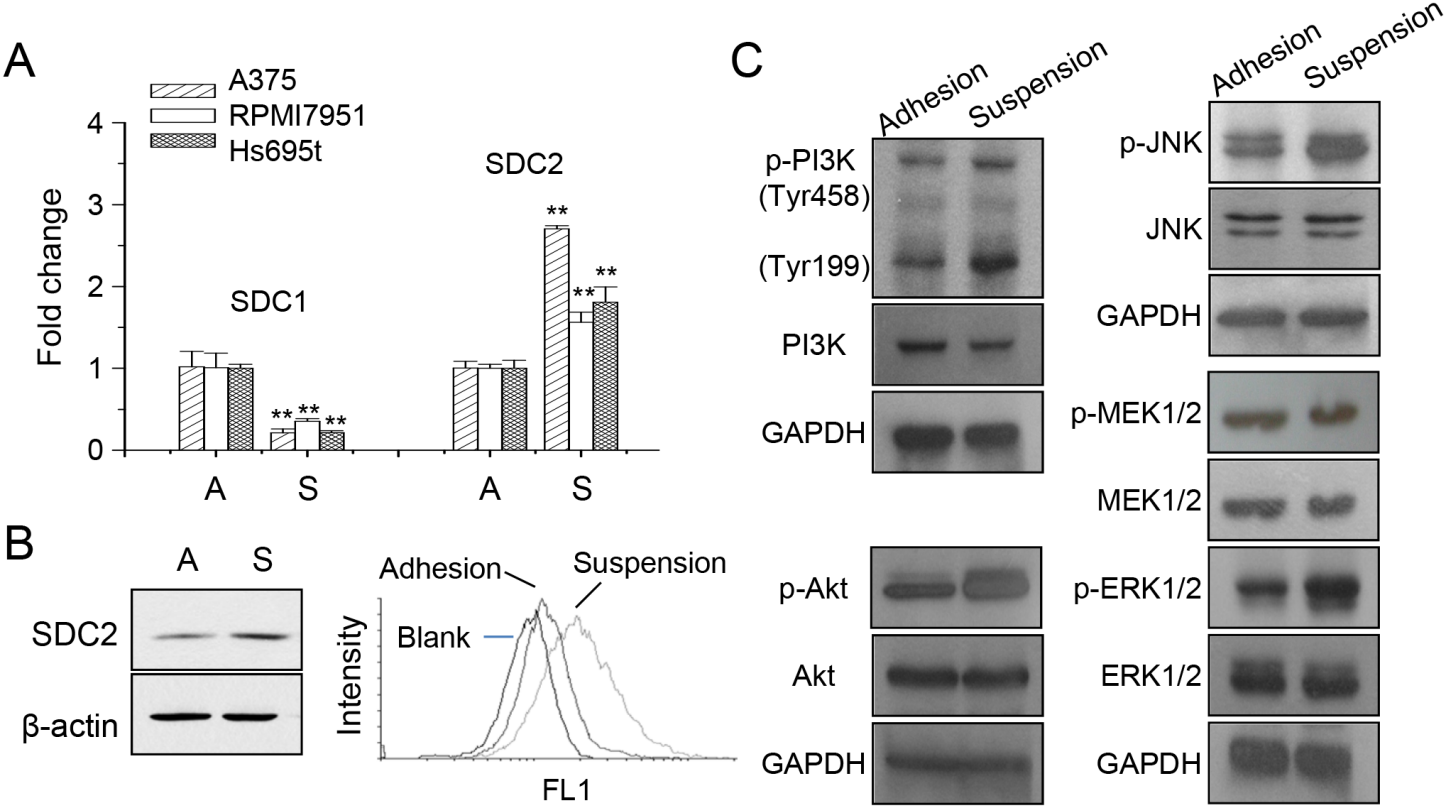
Increased SDC2 expression and kinase phosphorylation in melanoma cells under anchorage-independency **(A)** Expression of syndecan protein SDC1 and SDC2 under adhesion culture, A, or suspension culture, S, in different melanoma cells as analyzed by qPCR. Data were mean ±S.E. (n=3) **, *p* < 0.01. **(B)** SDC2 protein expression in A375 cells increased upon suspension as analyzed by western blot and flow cytometry. **(C)** Increased phosphorylation of PI3K, Akt, JNK, ERK, but not MEK, in suspended melanoma cells as examined by western blot.

Previously, we had shown the decreased expression of syndecan-1 (SDC1) was mediated by PKCδ that promoted loss of cell invasiveness for melanoma A375 cells under anchorage-independency [24]. The expression of SDC1 and SDC2 in different melanoma cells were examined (Figure 1A). The increase of SDC2 expression was more significant in melanoma A375 cells. We further examined the overall SDC2 protein expression and cell surface content by western blot and flow cytometry, respectively (Figure 1B). Under cell suspension, SDC2 protein level increased as well the level at cell surface. This suggested possible function of glycosaminoglycan-modified proteoglycan exhibited at plasma membrane. It had shown that differential EGFR-signaling promoted anchorage-independent growth of squamous carcinoma cells [25]. We examined the activation of signaling pathways under anchorage-independency by western blot. As seen in Figure 1C, phosphorylations of PI3K, Akt, JNK, MEK, and ERK1/2 increased upon cell suspension. This was consistent with other observation that several signaling pathways were activated upon anoikis resistance [26, 27].

### SDC2 expression was mediated by PKCδ under anchorage-independency

**Figure 2:**
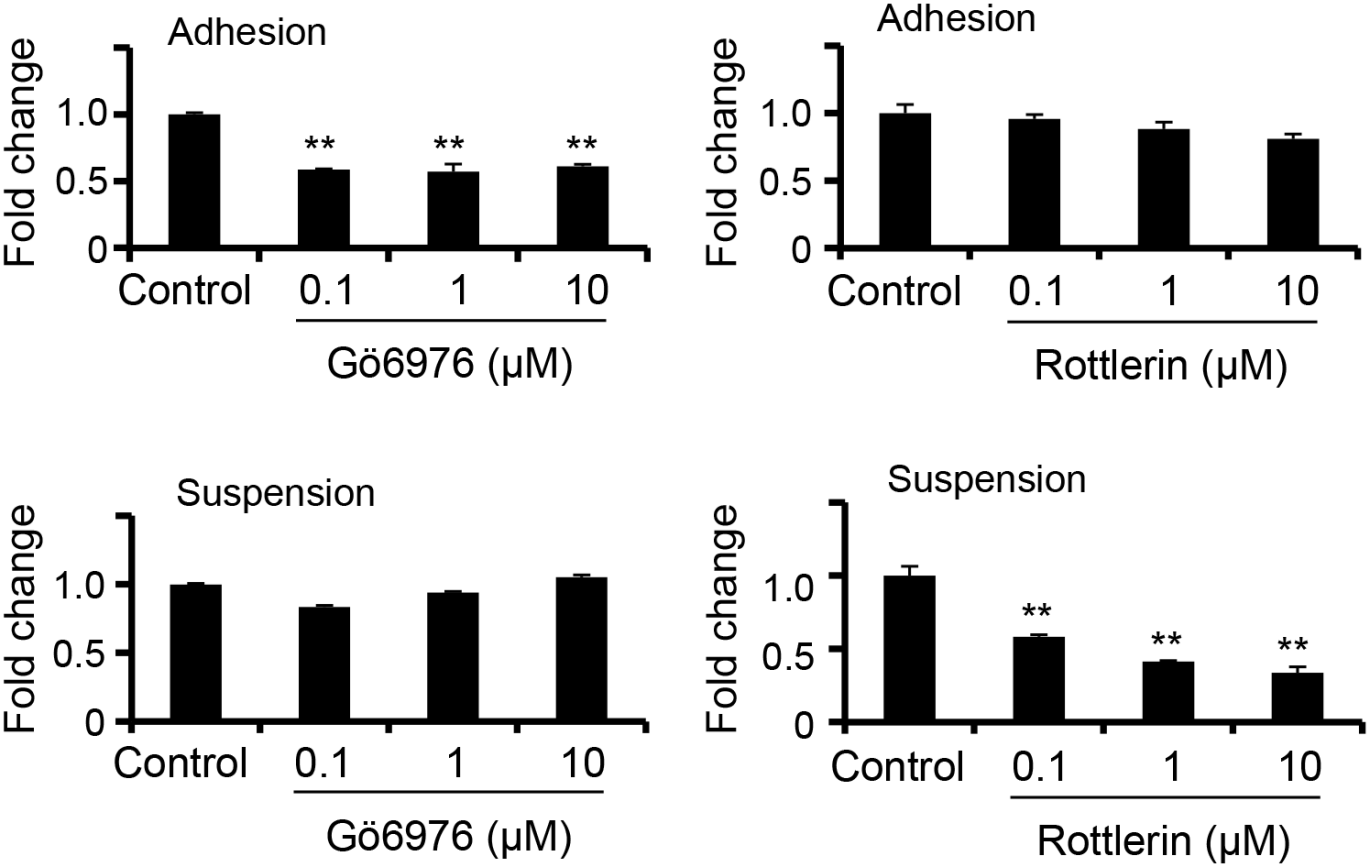
PKCδ-mediated SDC2 upregulation under anchorage-independency Gö6976 (PKCα/β inhibitor) suppressed SDC2 mRNA expression only upon cell adhesion, while Rottlerin (PKCδ inhibitor) suppressed SDC2 mRNA expression only upon cell suspension as examined by qPCR. Data were mean ±S.E. (n=3) **, *p* < 0.01.

Previously, we had demonstrated that attachment impairment contributed to PKCδ activation and downregulation of SDC1 expression [24]. We also examined whether the increased SDC2 expression in suspended melanoma cells was also mediated by PKCδ activation. Specific inhibitor on PKCα/β, Gö6976 [28], and inhibitor on PKCδ, Rottlerin [28], were used to treat cells and the level of SDC2 expression was characterized by qPCR. As seen in Figure 2, PKCα/β inhibitor, but not PKCδ inhibitor, reduced SDC2 expression in adherent melanoma cells. In contrast, SDC2 expression in suspended melanoma cells was suppressed by PKCδ inhibitor, but not by PKCα/β inhibitor. This indicated that the PKC isoforms used in the regulatory mechanisms for SDC2 expression were changed upon anchorage-independency.

### SDC2 level enhanced activation of signaling pathway and cell mobility

**Figure 3:**
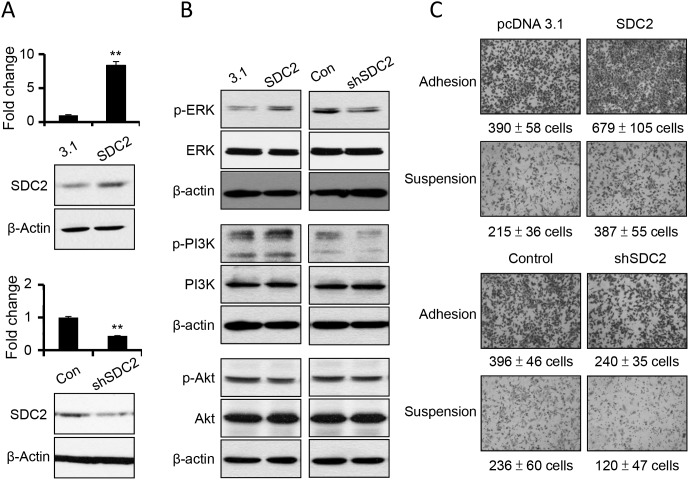
SDC2 overexpression increased ERK and PI3K activation and cell migration in melanoma cells **(A)** Transfection of SDC2 expression construct overexpress SDC2 mRNA and proteins, while transfection of SDC2-specific shRNA suppress those in melanoma cells as investigated by PCR and western blot. Data were mean ±S.E. (n=3) **, *p* < 0.01. **(B)** Phosphorylations of ERK and PI3K, but not of Akt increased upon SDC2 overexpression or decreased by SDC2-specific shRNA transfection. **(C)** SDC2-overexpressed melanoma cells showed higher migratory ability under adhesion or suspension. SDC2-supressed melanoma cells showed the opposite results. Data were mean ±S.E. (n=5).

In order to investigate the specific role of SDC2 upregulation upon anchorage-independency, we increased SDC2 expression level by overexpressing gene construct of SDC2 open reading frames, and suppressed SDC2 expression level by transfecting SDC2-specific shRNA. The level of SDC2 mRNAs and protein were characterized (Figure 3A). We further examined whether SDC2 level enhanced activations of signaling pathway and cell mobility. As seen in Figure 3B, increased SDC2 expression increased the levels of phospho-ERK and phospho-PI3K, while suppression of SDC2 expression decreased the levels of phospho-ERK and phospho-PI3K. The level of phosphor-Akt was not affected by overpression or suppression of SDC2 expression. This suggested the involvement of SDC2 in ERK activation and PI3K activation.

The change in SDC2 level affected cell invasiveness. As seen in Figure 3C, overexpression of SDC2 protein increased cell mobility while suppression of SDC2 expression decreased cell mobility at either adherent melanoma or suspended melanoma cells as investigated by transwell cell migration assay. This results were consistent with previous literature that SDC2 increased migratory potential of melanoma cells [19]. Previously, we demonstrated that downregulation of SDC1 inhibited cell adhesion and invasiveness [24]. In this study, we suggested SDC2 overexpression would enhance cell migration, however, suspended melanoma cells with higher SDC2 expression showed characteristics of invasiveness loss [24]. It was explained that SDC1 downregulation overwhelm the effect of SDC2 upregulation by suppression of laminin-binding and matrix metalloproteinase-2 secretion that led to loss of cell invasiveness [24].

### Increased SDC2 level promoted chemosensitivity upon cell suspension through PI3K activation

Several literatures suggested that SDC2 promoted cell apoptosis or increased chemosensitization [21–23, 29]. Since SDC2 expression were upregulated in anoikis-resistant melanoma cells upon suspension, we examined whether increased chemosensitization was also presented.

**Figure 4:**
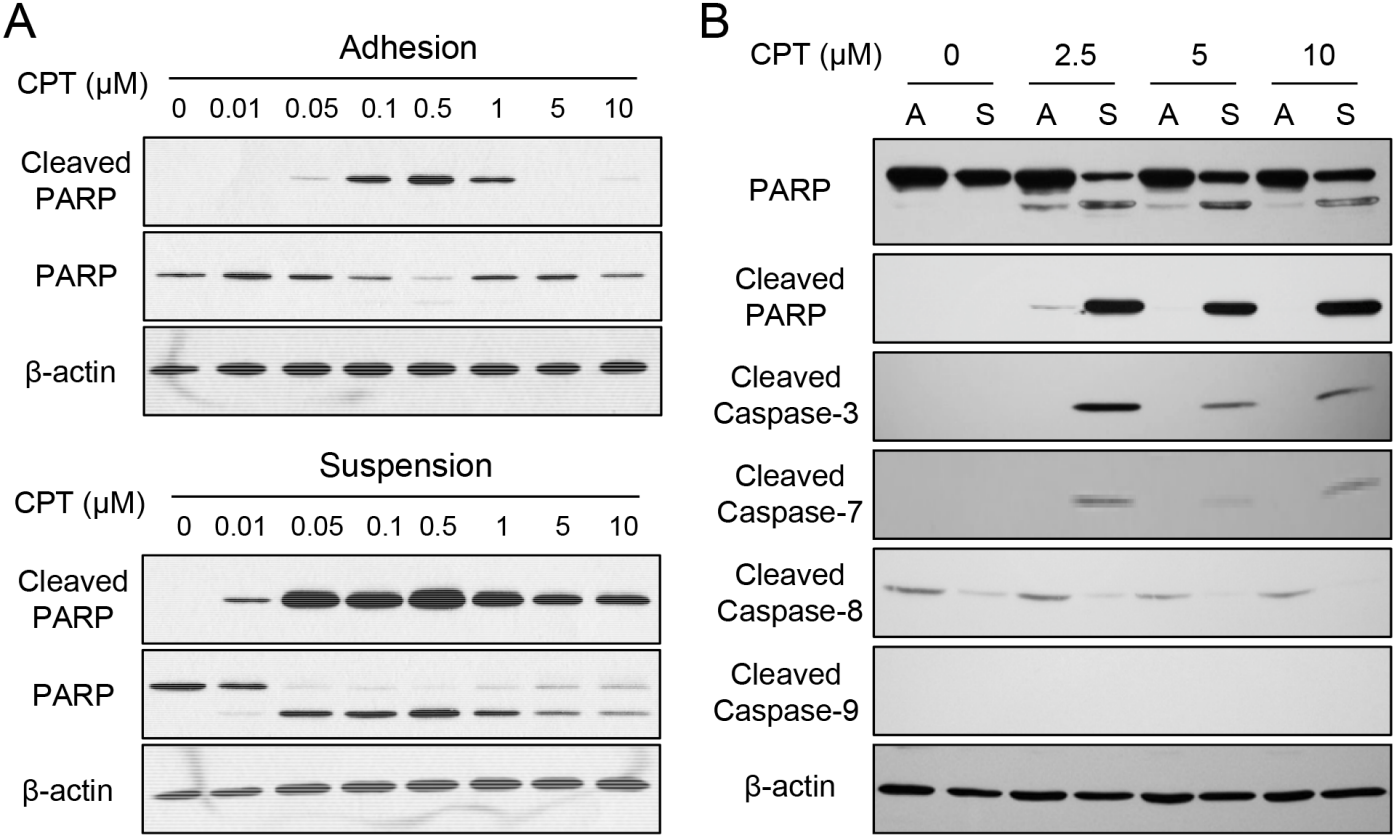
Enhanced chemosensitivity in suspended melanoma cells was associated with caspase-7 **(A)** Suspended melanoma cells were more sensitive to CPT treatments as revealed by apoptosis marker, PARP cleavage. **(B)** Enhanced CPT-induced apoptosis in suspended melanoma cells were associated with elevated caspase-7 cleavage / activation as seen by western blot.

As seen in Figure 4A, treatment of camptothecin (CPT) in adherent cells exhibited cell apoptosis in the concentration ranging from 0.1 to 0.5 μM as revealed by the presence of cleaved poly ADP ribose polymerase (PARP), a typical hallmark of caspase activation and cell apoptosis. However, cleaved PARP was seen by treating as low at 0.01 μM CPT for suspended melanoma cells (Figure 4A). Higher amount of cleaved PARP was seen in suspended melanoma cells by treatments at the same CPT concentrations (0.1 to 0.5 μM) than those in adherent cells. We did test other chemoagents (sorafenib and carmustine) and other melanoma cells, that enhanced chemosensitivity was also seen for melanoma cells under suspension (data not shown). This indicated the chemosensitivity of melanoma cells increased under cell suspension.

PARP is the downstream substrate of caspase-3 or caspase-7 that is activated by multiple activation routes of other caspase isoforms. As seen in Figure 4B, activations of different caspase isoforms were examined after CPT treatment at high concentrations for adherent melanoma (labeled as A) and suspended melanoma cells (labeled as S). With the presence of cleaved PARP under cell suspension, cleaved caspase-3 was also seen at suspended melanoma cells upon CPT treatment. No sign of caspase-8 or caspase-9 activation, but the cleavage of caspase 7 were seen upon CPT treatment in suspended melanoma. This suggested caspase-7 activation was associated with chemosensitization of melanoma cells upon suspension.

**Figure 5:**
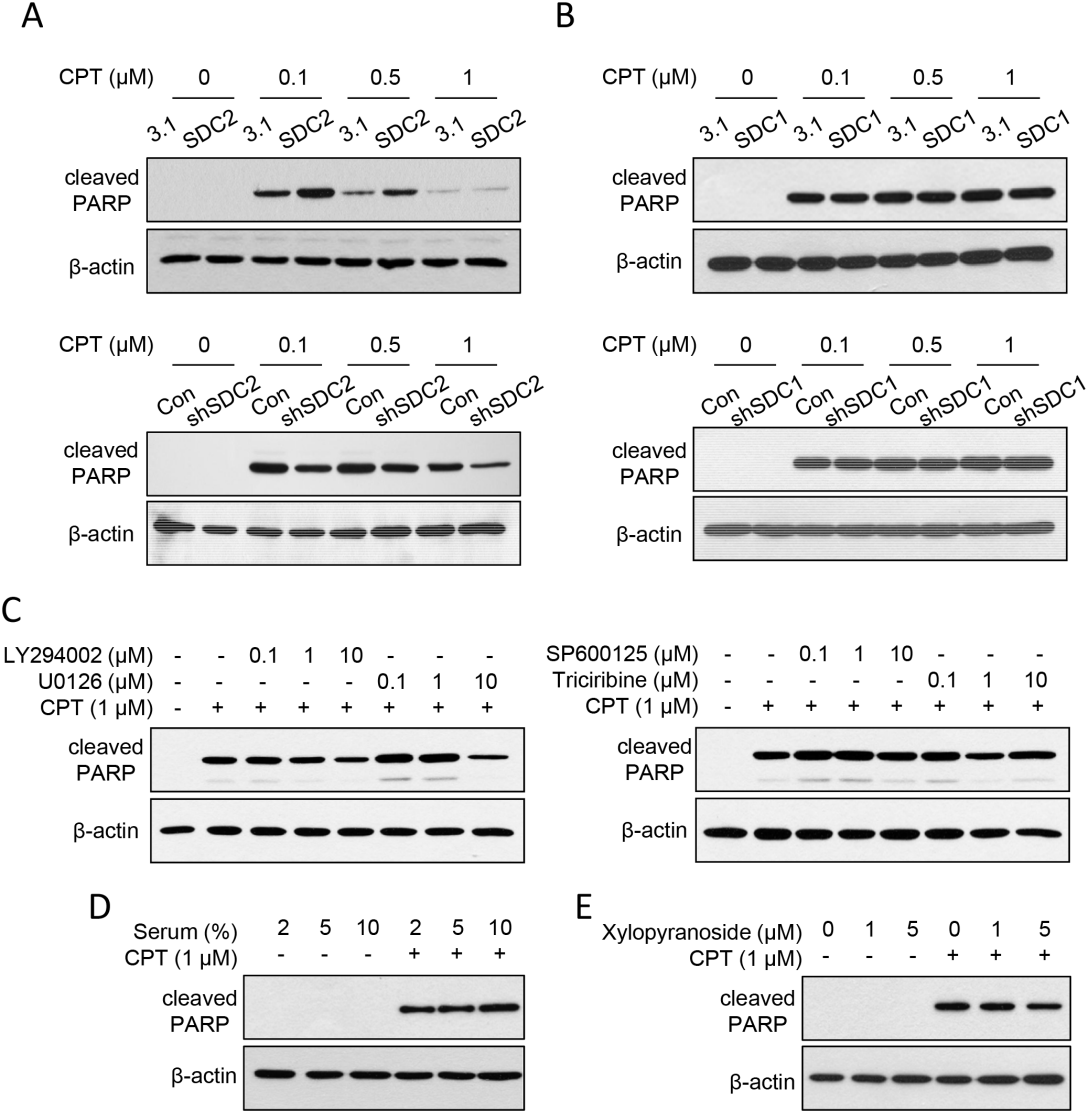
SDC2 promoted chemosensitivity through PI3K and ERK pathways through glycosaminoglycan chains **(A)** SDC2 overexpression sensitized CPT-induced apoptosis in suspended melanoma. Suppression of SDC2 expression reduced CPT-induced apoptosis. ECL detection was film-exposed for 1 min (for SDC2 overexpression) or 3 min (for SDC2 suppression). **(B)** SDC1 overexpression or suppression did not affect the level of CPT-induced apoptosis in suspended melanoma. ECL detection was both film-exposed for 3 min. **(C)** Effect of LY294002 (PI3K inhibitor), U0126 (MEK/ERK inhibitor), SP600125 (JNK inhibitor), and triciribine (Akt inhibitor) on CPT-induced apoptosis in suspended melanoma. Decreased PARP cleavage was observed by treatment of PI3K inhibitor and ERK inhibitor at higher concentration. **(D)** Lower serum level protected suspended melanoma cells from CPT-induced apoptosis. **(E)** Treatment of glycosaminoglycan-synthesis inhibitor, xylopyranoside, suppressed CPT-induced apoptosis as seen by reduced PARP cleavage.

After increasing the SDC2 expression level, we examined its effect on chemosensitization upon cell suspension. For suspended melanoma cells, SDC2 overexpression increased the amount of cleaved PARP, while suppression of SDC2 expression decreased the amount of cleaved PARP (Figure 5A). We also overexpressed the SDC1 protein, which belonged to SDC superfamily and was downregulated in melanoma A375 cells under anchorage-independency [24]. However, no difference in cleaved PARP level by SDC1 overexpression or suppression (Figure 5B). This suggested that SDC2 protein specifically enhanced chemosensitization in suspended melanoma cells.

### PI3K activation was associated with SDC2-mediated increase of chemosensitivity

In further, we investigated whether SDC2-mediated chemosensitization was associated with signaling pathway activation. As shown in Figure 5C, the PI3K inhibitor (LY294002) reduced PARP cleavage, which indicated PI3K activation might promote chemosensitization. MEK/ERK inhibitor (U0126) was effective to reduce only at higher concentration (10 μM). The effect of JNK inhibitor (SP600125) and Akt inhibitor (triciribine) were marginally effective that no significant change was observed (Figure 5C). This suggested that activation of PI3K and ERK pathways under cell suspension enhanced chemosensitivity (Figure 1C), which were mediated by SDC2 upregulation (Figure 1A, 1B, and 3B).

SDC2 is the heparan-sulfate proteoglycan that mediates cell activity through its glycosaminoglycan binding with many cytokines or growth factors. We investigated whether increased chemosensitivity was associated with serum concentration and the presence of glycosaminoglycan chain. As seen in Figure 5D, decrease in serum concentration suppressed CPT-induced apoptosis as determined by less cleaved PARP. Solely reducing the serum concentrations did not affect cell apoptosis. These results implied the growth factors in the serum promoted CPT-induced apoptosis in suspended melanoma cells.

To examine whether glycosaminoglycan chains on SDC2 were involved in CPT-induced apoptosis, we inhibited glycosaminoglycan synthesis by pretreatment of xylopyranoside [30]. As shown in Figure 5E, inhibition of glycosaminoglycan synthesis suppressed CPT-induced apoptosis in suspended melanoma. These results suggested SDC2 promoted chemosensitivity through its glycosaminoglycan binding sites for serum proteins that would activate PI3K or ERK pathways. Upregulation of SDC2 in melanoma cells under anchorage independency enabled higher sensitivity to lower dose of chemoagents, which might prevent spreading of malignant melanoma under suspension.

## DISCUSSION

Anchorage-independent survival was one of key features in malignant tumor cells [27]. Activation of specific signaling pathways, such as PI3K pathway, were associated with their survival under suspension [31–33] or resistance to apoptosis [27, 34]. In this paper, we showed the activation of PI3K, Akt, ERK, and JNK upon anchorage-independency, which were associated with enhanced sensitivity of suspended melanoma cells toward chemoagents. It had been shown that suppression of NF-kB activation to enhance chemosensitivity by imidazoline in leukemia T cells responsive to camptothecin [35] or by curcumin in esophageal adenocarcinoma responsive to 5-fluorouracil or cisplatin [36]. Inhibition of the PI3K/Akt pathway by LY294002 increases the chemosensitivity of gastric cancer cell toward vincristine [37]. Our results demonstrated the activation of PI3K pathway promoted chemosensitivity of suspended melanoma cells. We suggested the PI3K pathway might act as double-edged sword that promote cell survival and enhanced damage by chemoagents under anchorage-independency.

Heparan sulfate chains in SDC2 proteoglycans act as binding acceptors for several growth factors or chemokines. We demonstrated the elevated serum concentration promoted CPT-induced apoptosis through the glycosaminoglycan chains, that would activate PI3K or ERK pathways that enhanced chemosensitivity of melanoma cells under suspension. This is an interesting phenomenon but was in contrast with other observations, at which serum-depletion might induce cell apoptosis or enhance chemosensitivity [38–40]. However, several literatures implied the growth factors or receptors promoted or involved in cell apoptosis [41–45].

Our observation could explain and support the current strategy of adjuvant chemotherapy to block the survival or to eliminate disseminated melanoma cells through low-dose administration of chemoagents after surgical removal and pre-metastatic stages in melanoma patients. Since enhanced chemosensitivity in the presence of serum was achieved by elevated SDC2 expression and pathway activation upon cell detachment, applications of specific chemoagents against melanoma in lower dosage would be effective to kill suspended melanoma cells. However, the clinical usage and proper doses need to be evaluated in advance.

## MATERIALS AND METHODS

### Cell culture and reagents

Human melanoma A375, RPMI7951, and Hs695t cells were all purchased from Bioresource Collection and Research Center (BCRC; Hsinchu, Taiwan) with authentication. Adherent culture were maintained on culture dish (Corning Incorporated Life Sciences, Tewksbury, MA, USA) containing DMEM medium supplemented with 10% (v/v) fetal bovine serum (Biological Industries Ltd., Cromwell, CT, USA). Suspended melanoma cells were established and cultured by impaired attachment of adherent melanoma cells according to the reported procedures [46, 47]. For inhibitor treatment, adherent or suspended melanoma cells in 2 × 10^5^ cells were incubated with inhibitors individually for 24 hrs in culture medium. The inhibitors used in this study: PKCα/β inhibitor (Gö6976), PKCδ inhibitor (rottlerin), PI3K inhibitor (LY294002), MEK/ERK inhibitor (U0126), JNK inhibitor (SP600125), Akt inhibitor (triciribine) were all purchased from Enzo Life Sciences Inc., Farmingdale, NY, USA. Xylopyranoside was purchased from Sigma-Aldrich, Co. LLC., St. Louis, MO, USA.

### Construct and cell transfection

Cloning of *SDC2* coding sequence was done by polymerase chain reaction (PCR) using Ampliqon III UniPol enzyme mixture, melanoma cDNA pools, and SDC2 gene-specific primers (forward primer, cctgatgaattcatgcggcgcgcgtggatcctgc; reverse primer, cctgattctagattacgcataaaactccttagtaggtgc). The PCR product was digested by *EcoR*I or *Xba*I and ligated into pCDNA3.1 vector predigested with the same enzymes. Construction of SDC1 overexpression plasmid was as described [24]. The plasmids expressing shRNAs against *SDC2* and *SDC1* gene were purchased from the National RNAi Core Facility located at Institute of Molecular Biology / Genomic Research Center, Academia Sinica, Taiwan. Cells were transfected using TurboFect transfection reagent (Thermo Fisher Scientific Inc., Pittsburgh, PA, USA) according to the manufacturer’s instruction. The transfected cells were selected and enriched under growth medium containing 0.5 mg/mL hygromycin B (for overexpressed cells) or 5 μg/mL puromycin (for shRNA-transfected cells).

### Polymerase chain reactions and statistical analysis

The levels of mRNA in cultured cells were analyzed by reverse-transcription PCR and quantified by quantitative real-time PCR (qPCR). The forward and reverse primers to analyze SDC1 or SDC2 expression used in PCR were listed as referred [24]. The cDNAs were synthesized by MMLV HP reverse transcriptase (Epicentre, Madison, WI, USA) using freshly prepared RNA as PCR template. Quantitative real-time PCR were performed using VeriQuest Fast SYBR green qPCR reagent (Affymetrix Inc., Santa Clara, CA, USA) in a StepOne Plus real-time PCR system (Thermo Fisher Scientific Inc., Pittsburgh, PA, USA). The 2^-ΔΔCT^ method was used to determine the relative gene expression using GAPDH as control. The *p*-value of < 0.05 or < 0.01 was statistically significant and was indicated in each figures.

### Western blot and antibodies

Western blot was performed according to standard protocol. The concentrations of antibodies used for western blot analysis were according to manufacturer's instructions. The primary antibodies against SDC2 protein was obtained from Thermo Fisher Scientific Inc., Pittsburgh, PA, USA. The primary antibodies against phospho-PI3K (p85-Tyr458/p55-Tyr199), PI3K, phospho-Akt (Ser473), Akt, phospho-JNK (Thr183/Tyr185), JNK, phospho-MEK (Thr286), MEK, phospho-ERK (Thr202/Tyr204), ERK, and the antibody kit for apoptosis-related protein (PARP, cleaved PARP, caspase-3, caspase-7, and caspase-9) were all purchased from Cell signaling Inc., Danvers, MA, USA. The primary antibody against anti-caspase-8, GAPDH, and β-actin were purchased from GeneTex Inc., Hsinchu, Taiwan.

### Flow cytometry analysis

SDC2 expression analyzed by flow cytometry was performed accordingly [48]. 1 × 10^5^ cells were incubated with 1 μg anti-SDC2 primary antibody (Thermo Fisher Scientific Inc., Pittsburgh, PA, USA) overnight at 4°C, and 0.5 μg FITC-labeled secondary antibody at room temperature for 1 hr. The labeled cells were then analyzed by Partec flow cytometer ML (Partec North America, Inc., Swedesboro, NJ, USA).

### Transwell migration assay

For transwell migration assay, culture insert (8 um pore size; BD Company, Franklin Lakes, NJ, USA) were precoated with matrigel (BD Company, Franklin Lakes, NJ, USA). 1×10^5^ cells (in DMEM medium with 1% (v/v) FBS) were applied into the insert. The bottom wells contained 600 uL of culture medium with 10% (v/v) FBS, and were left un-agitated in the cell incubator until observation.

## Abbreviations

PCR: polymerase chain reaction
PI3K: phosphoinositide-3-kinase
ERK: extracellular signal–regulated kinases
MEK: Mitogen-activated protein kinase kinase
JNK: c-Jun N-terminal kinases
PKC: protein kinase C
SDC1: syndecan-1
SDC2: syndecan-2
CPT: camptothecin
PARP: poly ADP-ribose polymerase
